# Gestational Weight Gain and Exposure of Newborns to Persistent Organic Pollutants

**DOI:** 10.1289/ehp.1306758

**Published:** 2014-05-02

**Authors:** Esther Vizcaino, Joan O. Grimalt, Berit Glomstad, Ana Fernández-Somoano, Adonina Tardón

**Affiliations:** 1 Department of Preventive Medicine and Public Health, University of Oviedo, Asturias, Spain; 2 Department of Environmental Chemistry, Institute of Environmental Assessment and Water Research (IDÆA-CSIC), Barcelona, Catalonia, Spain; 3 Spanish Consortium for Research on Epidemiology and Public Health (CIBERESP), Instituto de Salud Carlos III, Madrid, Spain

## Abstract

Background: Exposure to persistent organic pollutants (POPs) during fetal development can increase the risk of adverse health effects during childhood. Maternal characteristics and physiological changes during gestation, such as gestational weight gain (GWG), may have an influence in the overall burden of POPs in neonates. However, the associations between GWG and POP concentrations are still not well established.

Objective: We examined the association of GWG with cord serum POPs concentrations after adjusting for prepregnancy maternal body mass index (BMI) and other potential determinants of the transfer of POPs into newborns. The GWG values were evaluated after grouping by the reference guidelines of the Institute of Medicine (IOM).

Methods: We measured levels of 14 organochlorine pesticides, 7 polychlorobiphenyls (PCBs), and 14 polybrominated diphenyl ethers (PBDEs) in 325 cord serum samples from a Spanish birth cohort. Multivariable models were used to estimate associations of GWG, prepregnancy BMI, and other maternal determinants on cord serum concentrations of POPs.

Results: Neonatal concentrations of POPs were inversely associated with GWG after adjustment for age, prepregnancy BMI, educational level, and fish consumption. On average, neonates of women with IOM-recommended GWG have lower POP concentrations than do neonates of mothers with inadequate GWG.

Conclusions: The present findings suggest an association between neonatal exposure to POPs and inadequate GWG during pregnancy. Encouraging pregnant women to meet the recommended IOM guidelines for GWG may reduce the accumulation of POPs in newborns.

Citation: Vizcaino E, Grimalt JO, Glomstad B, Fernández-Somoano A, Tardón A. 2014. Gestational weight gain and exposure of newborns to persistent organic pollutants. Environ Health Perspect 122:873–879; http://dx.doi.org/10.1289/ehp.1306758

## Introduction

Increasing epidemiological evidence suggests that exposure to stressors during early life has an influence on later development of some diseases (Boekelheide et al. 2012). *In utero* exposure to environmental pollutants is of particular concern because the immune and metabolic systems of fetuses are still in formation and more vulnerable to the adverse effects of toxic chemicals. Persistent organic pollutants (POPs) such as DDTs, hexachlorocyclohexanes (HCHs), pentachlorobenzene (PeCB), hexachlorobenzene (HCB), polychlorobiphenyls (PCBs), and polybrominated diphenyl ethers (PBDEs) are widespread toxic environmental pollutants that have been used extensively as pesticides, flame retardants, hydraulic fluids, or in other applications (Stockholm Convention on Persistent Organic Pollutants 2004). Nowadays their synthesis is severely restricted or forbidden, but they are still found in the environment and in human tissues (Carrizo et al. 2006; Grimalt et al. 2001; Simonich and Hites 1995) as consequence of their high persistence and liposolubility. *In utero* exposure to background POP levels has been associated with evidence of endocrine disruption (Herbstman et al. 2008; Lopez-Espinosa et al. 2010), neurodevelopmental disorders (Forns et al. 2012; Herbstman et al. 2007b; Jacobson and Jacobson 2003), and immunosuppression (Hertz-Picciotto et al. 2008).

Gestational weight gain (GWG) is itself a potential influence on the growth and health of the fetus and later outcomes during childhood and adulthood (Viswanathan et al. 2008). Inadequate GWG has been associated with low birth weight and preterm birth (Han et al. 2011), whereas excessive GWG has been associated with childhood obesity (Oken et al. 2007). GWG might be related to prepregnancy body mass index (BMI) (Dietz et al. 2006). In view of the increasing prevalence of overweight and obesity among childbearing women, the Institute of Medicine (IOM) has provided specific recommendations for GWG, and has stated the need for research on possible relationships between environmental exposures and GWG (IOM 2009). Associations between prenatal POP exposures and birth weight (Govarts et al. 2012; Lopez-Espinosa et al. 2011), early obesity (Valvi et al. 2012), and preterm birth (Bergonzi et al. 2011) have been reported. Given these findings, further knowledge is needed to assess the importance of GWG on newborn POP concentrations.

POPs are able to cross the placenta during pregnancy (Sala et al. 2001). Thus, children already come to life with an initial body burden of these compounds, which depends partly on anthropometric and sociodemographic maternal characteristics such as age, prepregnancy BMI, GWG, educational level and social class, obstetric and lactational history, and diet (Glynn et al. 2007; Sarcinelli et al. 2003; Vizcaino et al. 2010; Wolff et al. 2005b).

Modifications of serum POP levels have been related to weight changes in the general population (Lim et al. 2011; Wolff et al. 2005a) or in obese individuals (Chevrier et al. 2000). Weight loss increases circulating concentrations of these compounds because they are stored mainly in adipose tissue and are released during lipid mobilization. In contrast, weight gain tends to dilute POP levels in serum. Very little is known about the influence of GWG on fetal POP concentrations, despite the substantial changes in maternal weight during pregnancy. One study of newborns from Baltimore, Maryland (*n* = 297) (Herbstman et al. 2007a) did not show consistent associations between GWG and cord serum PBDEs and PCBs levels within the study population. PCBs were also considered in a similar study of mothers from Lake Ontario (*n* = 193) (Stewart et al. 2000), and the results did not show any significant association. None of these previous studies considered the IOM GWG recommendations or investigated potential modifier effects of prepregnancy BMI on GWG in a wide range of POPs.

We aimed to investigate the potential influence of GWG on newborn POP exposures adjusting for other potential determinants, including GWG as a continuous measure and GWG categories defined according to the IOM guidelines (IOM 2009).

## Material and Methods

*Study population.* The study sample was drawn from a cohort of mothers and their newborns, the Asturias cohort, established by the University of Oviedo, as part of the INMA [INfancia y Medio Ambiente (Environment and Childhood)] project (Guxens et al. 2012). A total of 494 pregnant women were recruited (May 2004–June 2007). Maternal serum samples were collected during the first trimester of gestation (median, 12 weeks; range, 10–13 weeks). Deliveries took place between October 2004 and February 2008 at San Agustín Hospital (Avilés, Spain). Three hundred twenty-five cord blood samples were successfully collected by hospital staff assisting at 485 childbirths within the cohort. POP concentrations in 325 mother–umbilical cord serum paired samples were analyzed. The characteristics of the mothers with sampling were comparable with those of the rest of the cohort (data not shown). The study protocol was approved by the Ethics Committee of San Agustín hospital, and informed consent was provided by every participant.

*POP analyses.* The laboratory methods and quality control procedures for the analysis of organohalogen compounds have been described elsewhere (Grimalt et al. 2010; Vizcaino et al. 2009). Further description of the analytical methods is provided in Supplemental Material (see Supplemental Material, Analytical Methods, p. 2).

*Gestational weight gain.* GWG was defined as the difference between the last recorded weight during pregnancy and the mother’s self-reported prepregnancy weight. To avoid possible heteroscedasticity and nonlinearity effects, GWG was calculated from weekly GWG rates (wGWG) during pregnancy, which were calculated for each week based on differences between weight measurements from prenatal visit records and the self-reported prepregnancy weight, divided by the gestational age at each measurement. The mean (± SD) number of weight measurements per mother was 6.5 ± 2.7. Self-reported prepregnancy body weight was replaced with the first clinical record of body weight if it was measured before 8 weeks of gestation, and the second measure was not recorded before 15 weeks (*n* = 1) (Nielsen et al. 2006). Missing or implausible self-reported prepregnancy weight (when wGWG was *>* 1.5 kg/week or *<* –1 kg/week) was imputed by linear extrapolation when two GWG were recorded before 15 gestational weeks (*n* = 4).

Total rate of weekly GWG (twGWG) was calculated as the difference between the last weight at the end of pregnancy and the self-reported prepregnancy weight divided by gestational age. For women whose last weight measurement was recorded during 37.5–42 weeks of gestation (*n* = 130; see Supplemental Material, Table S1), we used the measurement taken closest to 39 weeks (wGWG_39_). This measurement was not necessarily the last, because GWG stalls at the end of pregnancy.

For 194 women who did not have a body weight measurement recorded during 37.5–42 weeks of gestation, we calculated twGWG_39_ from their last measured weight. Specifically, for woman *i* and week *k*:

twGWG*^i^*_39_ = mean(wGWG_39_) + {[twGWG*^i^_k_* – mean(wGWG*_k_*)] × [SD(wGWG_39_)/SD(wGWG*_k_*)]}. [1]

The mean(wGWG_39_), mean(wGWG_k_), SD(wGWG_39_), and SD(wGWG_k_) values were obtained from the wGWG distribution of a pooled INMA cohort encompassing pregnant women from Asturias, Gipuzkoa, Sabadell, and Valencia (*n* = 2,413). The wGWG distributions in the Asturias and the reference INMA cohorts were similar involving higher wGWG and lower heteroscedasticity (SD) at higher gestational age (see Supplemental Material, Tables S1 and S2), which is in agreement with previous studies (Dietz et al. 2006; Kleinman et al. 2007; Nohr et al. 2009).

Finally, total GWG was calculated as the product of gestational age in weeks and twGWG_39_. This variable was modeled as a continuous variable (total kilograms) and as a categorical variable (recommended, inadequate, or excessive) as defined by the IOM (Rasmussen et al. 2009) according to prepregnancy BMI. Specifically, recommended GWG is 11.25–15.75 kg for women classified as having normal prepregnancy BMI (as defined by the World Health Organization 2014), and as 12.60–18.00 kg, 6.75–11.25 kg, and 4.95–9.00 kg for women classified as underweight, overweight, and obese, respectively.

*Other variables.* Gestational age was calculated from the date of the last menstrual period reported at recruitment and was confirmed using ultrasound examination in week 12 of gestation. If reported gestational age and ultrasound determination differed by > 6 days (12.9% of participants), it was recalculated from the crown–rump length using an early ultrasound measurement (Westerway et al. 2000).

Age, prepregnancy BMI, parity, education level, social class, fish intake, and previous breastfeeding history were considered potential confounding factors because of their possible associations with GWG and POP serum concentrations (Glynn et al. 2007; Sarcinelli et al. 2003; Vizcaino et al. 2010; Wolff et al. 2005b). Pregnant women completed two detailed in-person questionnaires (weeks 10–13 and 28–32) and provided information on parity, age, social class [defined according to a widely used Spanish adaptation of the international ISCO-88 (International Standard Classification of Occupations) coding system (Vrijheid et al. 2012)], education level, prepregnancy BMI (based on measured height at recruitment and self-reported prepregnancy weight), and previous breastfeeding history. Dietary information was collected from a validated semiquantitive food frequency questionnaire of 101 food items (Vioque et al. 2013) and focused on dairy products, meat, vegetables, fruits, and fish. Total fish consumption included lean fish, oily fish, canned tuna fish, seafood as well as processed fish, mixed fried fish, and dried or smoked fish.

*Data analysis.* POP concentrations were expressed in nanograms per milliliter of serum and in nanograms per gram serum lipid. Univariate and multivariable analyses were performed using both concentrations, providing the same results; hence, only the results for serum lipid concentrations are shown. Values of half detection limit were assigned when measured analyte concentrations were below the limit of detection (LOD).

Nonparametric tests were used for bivariate analysis. Associations between POP concentrations and potential sociodemographic and dietary predictors and were assessed with Spearman rank correlations, Mann–Whitney *U-*test, and Kruskal–Wallis test, with *p*-values adjusted to control for false discovery rate (Benjamini and Hochberg 1995). Multivariable models were constructed with natural log (ln)–transformed POP concentrations as dependent variables. Linear regression was used for the organochlorine compounds (OCs) that were *>* LOD in 90% of cord and maternal serum samples [β-HCH, HCB, 4,4´-DDT, 4,4´-DDE, and three PCBs (PCB-153, PCB-138, and PCB-180)] (see Supplemental Material, Tables S3 and S4). The concentrations of PCBs 138, 153, and 180 in cord serum were highly correlated (Spearman *r >* 0.9), so in addition to individual PCBs we estimated associations with summed PCBs (ΣPCBs) calculated as PCB-153 + PCB-138 + PCB-180. We used Tobit regression models to estimate associations with BDEs (BDE-47, BDE-99, BDE-153, and BDE-209) to obtain maximum-likelihood estimates in the presence of censored values because the majority of PBDE concentrations were *<* LOD. BDE-47, BDE-99, BDE-153, and BDE-209 were strongly correlated (Spearman *r >* 0.7), and for this reason they were also considered jointly as summed total (ΣPBDEs) in the analyses.

POPs with concentrations *>* LOD in < 30% of cord serum samples were not evaluated further (PeCB, γ-HCH, δ-HCH, α-HCH, 2,4´-DDT, 2,4´-DDE, 4,4´-DDD, 2,4´-DDD, PCB-28, PCB-52, PCB-101, PCB-118, BDE-17, BDE-28, BDE-66, BDE-71, BDE-85, BDE-100, BDE-138, BDE-154, BDE-183, BDE-190, and BDE-209).

Multivariable models were built starting with all variables that were associated with POPs in nonparametric analyses (*p <* 0.1). Variables that were not statistically significant predictors in the full model (with statistical significance defined as *p <* 0.05) were sequentially excluded from the models following a stepwise backward procedure, using the *F*-test of change in *R*^2^. Potential confounders were retained if the GWG coefficient changed by *>* 10% after removal. In addition, we adjusted all models for maternal serum POPs concentrations measured in samples collected during the first trimester, which were assumed to represent baseline maternal serum concentrations before important GWG has occurred.

Associations between GWG and ln-transformed POP concentrations were estimated using adjusted general additive models (GAMs). Nonlinear models (a cubic smoothing spline with 2–4 degrees of freedom) did not provide significant improvements in the descriptions of the relationships (*p >* 0.05), so linear models were used. Potential modification of associations between GWG and POPs concentrations by prepregnancy BMI was evaluated by stratified analysis. In addition, we estimated geometric mean POP concentrations in newborns according to IOM-categorized GWG using linear regression.

*Sensitivity analyses.* The precision of the twGWG_39_ prediction was evaluated by means of the coefficient of determination (*R*^2^) of the linear regression model between observed and predicted values. The systematic error was calculated from the bias of the prediction (see Supplemental Material, Table S5).

Maternal POPs concentrations in early pregnancy might be determined by other variables in the multivariable models, potentially resulting in problems of endogenity (see Supplemental Material, Figure S1). To address this issue, we repeated models adjusting for a variable that represents the proportional difference in umbilical cord POPs concentrations (*C_uc_*) relative to maternal concentrations (*C_m_*): *X* = (*C_uc_* – *C_m_*)/*C_m_*. In addition, we repeated models without adjusting for maternal serum POPs concentrations.

Models were also repeated after exclusion of preterm births (*n* = 9) because they are not included in the reference guidelines of IOM for GWG. We also performed a sensitivity analysis excluding women with no weight measurements after 28.5 weeks of gestation (*n* = 7).

STATA version 12 statistical software package (StataCorp, College Station, TX, USA) was used for the data analyses.

## Results

*Cord serum concentrations and maternal determinants.* The Supplemental Material, Tables S3 and S4, reports the cord serum and maternal concentrations of POPs quantifiable in > 30% of the samples.

The concentrations of organochlorine pollutants newborn cord serum samples were at least one order of magnitude higher than those of PBDEs (see Supplemental Material, Table S4). 4,4´-DDE was the pesticide found at highest concentration (median, 180 ng/g lipid) and was observed in 99.7% of the samples, followed by HCB (median, 50 ng/g lipid) in 97.6% of the samples. β-HCH was the dominant HCH isomer (median, 17 ng/g lipid; 90.5% of the samples). PCB-153 was the most abundant PCB congener (median, 47 ng/g lipid), followed by PCB-138 (median, 31 ng/g lipid) and PCB-180 (median, 27 ng/g lipid). Total BDEs in cord serum ranged from *<* LOD to 816 ng/g lipid, with a median of 3.9 ng/g lipid. BDE-153 was the most frequent congener (43%), followed by BDE-47 (36.5%). BDE-209 was detected in only 14.9% of the samples, but it was the BDE congener found at highest concentration when detected (mean, 4.1 ng/g lipid).

Mean maternal age was 31.4 ± 4.2 years. Of all the mothers, 40.2% had a university degree, which was about the same proportion (43.3%) of those only having completed secondary school (Table 1). Fifty-five percent of the mothers belonged to the lowest social groups (Table 1). Primiparous women constituted the largest group (63.1%), and the mean gestational age was 39.6 ± 1.4 weeks. Standardized BMI categories showed that 21.9% of the mothers were overweight and 6.5% were obese before pregnancy (Table 1). On average, gestational weight was 14.1 ± 5.2 kg (range, –2.9 to 34 kg). There was an inverse association between GWG and prepregnancy BMI (Spearman *r* = –0.16, *p <* 0.01). Most mothers did not conform to the IOM guidelines (Table 1). Fifty-five percent of overweight and obese women exceeded recommended GWG compared 37% of normoweight and underweight women. The proportions of women gaining less GWG than recommended ranges were 9%, 30%, 14%, and 20% for those underweight, normoweight, overweight, and obese.

**Table 1:** Concentrations (ng/g lipid) of the ∑PCBs (138, 153, and 180), 4,4´-DDT, 4,4´-DDE, HCB, β-HCH; and ∑BDEs (47, 99, 153, and 209) in newborn cord serum according to maternal characteristics (*n* = 325) (mean ± SD).

Characteristic	*n*	(%)	∑PCBs	∑BDEs	4,4´-DDT	4,4´-DDE	HCB	β-HCH
Age (years)			*r*^*a*^ = 0.54^##^	*r* = 0.05	*r* = 0.19^##^	*r* = 0.17^##^	*r* = 0.42^##^	*r* = 0.25^##^
< 30	105	32.2	74 ± 1.8	5.5 ± 2.5	27 ± 2.7	164 ± 2.5	33 ± 2.2	7 ± 6.0
30–34	136	41.7	122 ± 1.6	6.0 ± 2.2	33 ± 2.2	181 ± 2.5	55 ± 2.0	14 ± 4.1
≥ 35	85	26.1	148 ± 1.8	5.5 ± 2.5	37 ± 2.5	221 ± 2.2	74 ± 2.5	20 ± 4.1
Prepregnancy BMI (kg/m^2^)			*r* = –0.07^#^	*r* = –0.02	*r* = 0.07	*r* = 0.12	*r* = 0.25^##^	*r* = 0.12^#^
Underweight (< 18.5)	11	3.4	90 ± 2.2	8.2 ± 3.7	17 ± 2.2	90 ± 2.0)	18 ± 3.0	6.0 ± 5.0
Normal weight (18.5–25)	222	68.3	110 ± 1.8	5.5 ± 6.2	33 ± 2.5	181 ± 2.5	49 ± 2.2	12 ± 4.5
Overweight (25–30)	71	21.9	110 ± 1.8	5.5 ± 52.5	30 ± 2.2	200 ± 2.5	67 ± 2.2	15 ± 6.0
Obese (> 30)	21	6.5	82 ± 2.0	6.0 ± 2.5	40 ± 2.2	270 ± 2.2	49 ± 2.2	14 ± 3.7
Gestational weight gain (kg)			*r* = –0.26^##^	*r* = –0.11^#^	*r* = –0.18^#^	*r* = –0.18^#^	*r* = –0.22^##^	*r* = –0.23^##^
Inadequate	81	25	148 ± 1.8	6.7 ± 2.5	37 ± 2.5	221 ± 2.2	60 ± 2.2	20 ± 4.1
Recommended	108	33.3	110 ± 1.8	5.5 ± 2.2	33 ± 2.2	181 ± 2.5	49 ± 2.5	14 ± 4.5
Excessive	135	41.7	90 ± 2.0	5.5 ± 2.2	30 ± 2.5	164 ± 2.5	45 ± 2.2	10 ± 5.0
Parity
Primiparous	205	63.1	100 ± 1.8*	6.0 ± 2.5	33 ± 2.5	181 ± 2.5	49 ± 2.2	12 ± 4.5
Multiparous	120	36.9	122 ± 2.0	5.5 ± 2.2	33 ± 2.5	200 ± 2.2	55 ± 2.5	12 ± 5.5
Education
Up to primary	54	16.6	90 ± 2.0**	6.0 ± 2.7	40 ± 2.5	270 ± 2.5*	45 ± 2.2**	10 ± 5.5
Secondary	141	43.3	100 ± 1.8	5.5 ± 2.5	30 ± 2.5	164 ± 2.2	45 ± 2.5	11 ± 5.0
University	131	40.2	122 ± 1.8	5.5 ± 2.2	33 ± 2.5	181 ± 2.5	60 ± 2.2	15 ± 4.1
Socioeconomic status
I + II (highest)	75	23.2	134 ± 1.8**	5.5 ± 2.0	33 ± 2.2	181 ± 2.0	60 ± 2.0*	14 ± 4.1
III	72	22.2	122 ± 1.6	5.5 ± 2.2	33 ± 2.5	164 ± 2.2	60 ± 2.0	14 ± 4.1
IV + V (lowest)	177	54.6	90 ± 2.0	6.0 ± 2.5	33 ± 2.5	200 ± 2.7	45 ± 2.5	12 ± 5.0
Total fish consumption (g/day)
< 70	162	50	100 ± 2.0*	5.0 ± 2.2	33 ± 2.5	181 ± 2.5	45 ± 2.2*	10 ± 5.0
≥ 70	162	50	122 ± 1.8	6.7 ± 2.5	33 ± 2.5	181 ± 2.5	55 ± 2.2	15 ± 4.1
Breastfeeding^*b*^
Never	231	71.1	100 ± 1.8*	6.0 ± 2.5	33 ± 2.5	181 ± 2.0	49 ± 2.2	14 ± 4.5
< 16 weeks	47	14.4	122 ± 2.0	5.5 ± 2.2	37 ± 2.5	221 ± 2.2	49 ± 2.7	15 ± 5.0
≥ 16 weeks	48	14.7	134 ± 2.0	6.0 ± 2.2	33 ± 2.7	200 ± 2.5	55 ± 2.5	10 ± 6.0
^***a***^Spearman rho. ^***b***^Accumulated breastfeeding time as consequence of previous pregnancies. **p < 0.0001 and *p < 0.05 by Kruskall–Wallis test (categorical variables). ^#^*p* < 0.0001 and ^##^*p *< 0.05 for Spearman correlation (continuous variables). *p*-Values were adjusted using Benjamini and Hochberg’s method.								

*Bivariate analyses.* Significant associations between maternal determinants and some but not all measured POPs were observed. Negative correlations between all POPs analyzed and GWG were found (Table 1). When using the IOM weight categories, mothers with inadequate GWG had children with higher POP cord serum concentrations than mothers with recommended or excessive GWG. A positive association between prepregnancy BMI and HCB and β-HCH concentrations was observed.

All OC concentrations in cord serum but not PBDEs were statistically significantly associated with increasing maternal age (Table 1). Fish consumption was the only maternal dietary item associated with POPs, involving higher ΣPCBs, ΣBDEs, HCB, and β-HCH concentrations in newborns with maternal fish consumption above the median (Table 1).

ΣPCB and HCB levels were higher among neonates born to women with high education level (university grade) than to mothers with secondary or primary degrees; 4,4´-DDE levels were higher in neonates of women with primary education (Table 1). Concerning social class, higher levels of PCBs and HCB were found in children of mothers from the highest level (groups I and II), whereas no regular trend was observed for the other contaminants analyzed.

Significant associations with parity or previous maternal feeding history were observed only for ΣPCBs.

*Multivariable analyses.* The associations between GWG and POP concentrations in cord serum were also inverse in the multivariate models (Table 2). Specifically, GWG was inversely associated with ΣPCBs, 4,4´-DDE, and β-HCH, and, at the edge of significance, for HCB; but GWG was not associated with ΣBDEs or 4,4´-DDT. Concerning prepregnancy BMI, only cord serum HCB concentrations in underweight mothers showed a significant negative association, although all other OCs were lower in underweight women. Other statistically significant predictors of POP concentrations in cord serum were age (ΣPCBs, 4,4´-DDT, HCB, and β-HCH), education level (4,4´-DDE), and fish consumption (ΣPCBs, ΣBDEs, HCB, and β-HCH). Social class, parity, and breastfeeding were not significant predictors in the multivariable models.

**Table 2:** Adjusted associations [β (95%CI)]*^a^* for ln-transformed concentrations of POPs in cord serum and GWG, prepregnancy BMI, age, total fish consumption, education, and maternal concentrations at first trimester.

Predictor	∑PCBs	∑BDEs	4,4´-DDE	4,4´-DDT	HCB	β-HCH
Gestational weight gain (kg)	–0.01 (–0.02, 0.002)	–0.02 (–0.05, 0.008)	–0.016 (–0.03, –0.003)	–0.013 (–0.03, 0.005)	–0.012 (–0.03, 0.001)	–0.03 (–0.06, –0.003)
Prepregnancy BMI (kg/m^2^)
Underweight (< 18.5)^*b*^	–0.22 (–0.50, 0.06)	0.48 (–0.32,1.30)	–0.17 (–0.52, 0.17)	–0.43 (–0.92, 0.06)	–0.40 (–0.78, –0.02)	–0.08 (–0.88, 0.73)
Overweight (25–30)	–0.08 (–0.20, 0.05)	0.07 (–0.28, 0.44)	0.02 (–0.13, 0.17)	–0.19 (–0.42, 0.03)	0.13 (–0.03, 0.30)	–0.02 (–0.37, 0.34)
Obese (> 30)	–0.10 (–0.33, 0.12)	–0.07 (–0.63, 0.62)	0.22 (–0.04, 0.49)	0.06 (–0.31, 0.44)	–0.02 (–0.31, 0.26)	0.1 (–0.52, 0.72)
Age (years)	0.03 (0.01, 0.04)		0.01 (–0.02, 0.03)	0.04 (0.01, 0.06)	0.02 (0.006, 0.04)	0.02 (–0.02, 0.06)
Total fish consumption (g/day)	0.001 (0.00, 0.002)	0.004 (0.001, 0.007)			0.003 (0.0004, 0.003)	0.003 (0.0004, 0.006)
Education^*c*^
Up to primary			0.34 (0.16, 0.52)
University			–0.10 (–0.24, 0.04)
Maternal concentrations^*d*^	0.67 (0.54, 0.80)	0.04 (–0.17, 0.25)	0.74 (0.67, 0.82)	0.38 (0.29, 0.49)	0.66 (0.56, 0.76)	0.97 (0.76, 1.2)
^***a***^β coefficient per unit of log ng/g lipids. ^***b***^Reference group is normal weight. ^***c***^Reference group is secondary education. ^***d***^Measured at first trimester of pregnancy.						

When models were stratified by maternal prepregnancy BMI categories, associations between GWG and POPs appeared to differ across BMI groups (Table 3). Newborns of underweight women tended to have higher OC levels with higher GWG. This was opposite to newborns from normoweight, overweight, or obese women, who tended to have lower OC levels with higher GWG. In contrast, newborns of underweight women tended to have lower PBDE levels with higher GWG.

**Table 3:** Adjusted associations [β (95%CI)]*^a^* of ln-transformed POPs concentrations in cord serum and GWG according to prepregnancy BMI categories.

BMI category (kg/m^2^)	∑PCBs^*b*^	∑BDEs^*c*^	4,4´-DDE^*d*^	4,4´-DDT^*e*^	HCB^*b*^	β-HCH^*b*^
All population	–0.01 (–0.02, –0.002)	–0.02 (–0.05, 0.008)	–0.016 (–0.03, –0.003)	–0.013 (–0.03, 0.005)	–0.012 (–0.03, 0.001)	–0.03 (–0.06, –0.003)
Underweight (< 18.5)	0.042 (–0.06, 0.14)	–0.12 (–0.35, –0.003)	0.02 (–0.06, 0.11)	0.043 (–0.12, 0.21)	0.03 (–0.22, 0.28)	0.006 (–0.28, 0.29)
Normal (18.5–25)	–0.009 (–0.02, 0.003)	–0.02 (–0.04, –0.006)	–0.02 (–0.04, –0.005)	–0.015 (–0.04, 0.008)	–0.009 (–0.03, 0.006)	–0.02 (–0.06, 0.009)
Overweight (25–30)	–0.02 (–0.05,– 0.0003)	0.003 (–0.04, 0.04)	–0.009 (–0.03, 0.01)	0.0001 (–0.04, 0.04)	–0.024 (–0.05, 0.004)	–0.06 (–0.14, 0.02)
Obese (> 30)	–0.03 (–0.06, –0.0002)	0.02 (–0.04, 0.09)	–0.01 (–0.04, 0.02)	–0.03 (–0.09,–0.02)	–0.008 (–0.04, 0.03)	–0.05 (–0.13, 0.03)
^***a***^β coefficient per unit of log ng/g lipids. ^***b***^Adjusted for maternal age, total fish consumption, and maternal concentrations at first trimester of pregnancy. ^***c***^Adjusted for total fish consumption and maternal concentrations at first trimester of pregnancy. ^***d***^Adjusted for maternal age, total fish consumption, education, and maternal concentrations at first trimester of pregnancy. ^***e***^Adjusted for maternal age and maternal concentrations at first trimester of pregnancy.						

**Figure 1:**
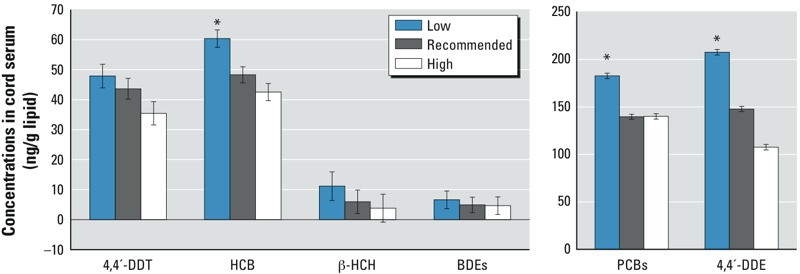
POP concentrations in newborns (adjusted geometric means and 95% CIs) from mothers in the different GWG categories according to the IOM guidelines (low, *n* = 81; recommended, *n* = 108; and high, *n* = 135). These results were adjusted for all variables that were significantly associated with POP concentrations in cord serum in the multivariable models. Geometric means according to GWG category are calculated assuming weighted average values of the other model covariates within each GWG category. *p < 0.05.

Grouping by the IOM categorical variable showed that the adjusted geometric mean cord blood POP concentrations from children of mothers with inadequate GWG were higher than those in children whose mothers had the recommended GWG (Figure 1). No differences were found between mothers with recommended or excessive GWG. These findings suggest that negative associations between POPs and GWG modeled as a continuous variable may have been driven by the positive association between POPs and inadequate weight gain during pregnancy.

*Sensitivity analyses.* The calculation method for twGWG_39_ showed a good agreement between predicted and observed twGWG_39_ values when there was a measure available in the third trimester. Estimated errors in the twGWG_39_ calculations decreased as the gestational week of the last weight measurement increased, with median relative percentage differences in estimated values relative to observed values of 6% for twGWG_36_ (*n* = 123), 7.9% for twGWG_33_ (*n* = 52), 9.5% for twGWG_30_ (*n* = 12), and 12.3% for twGWG_27_ (*n* = 7) (see Supplemental Material,Table S5). No significant changes were found in the results when preterm births (*n* = 9) were excluded (data not shown), or when data from mothers who did not have weight measurements after 28.5 weeks of gestation (*n* = 7) were excluded as well (see Supplemental Material, Table S6).

Associations between GWG and POPs were comparable when we excluded maternal prepregnancy BMI (data not shown), or excluded maternal POPs concentration as a model covariate, or modeled the relative difference in newborn concentrations compared with maternal concentrations (see Supplemental Material, Table S6). Overall, associations of GWG with individual PCB and PBDE congeners were consistent with associations of GWG with ΣPCBs and ΣBDEs (see Supplemental Material, Table S7, and Figures S2 and S3).

## Discussion

The concentrations of OCs found in this population were similar to those reported in cord serum from other representative European populations collected during the same time period (2004–2007) (Bergonzi et al. 2009; Koppen et al. 2009; Vizcaino et al. 2010) and lower than those found in European studies of earlier periods (1996–2000) (Carrizo et al. 2006; Sala et al. 2001). The PBDE concentrations were similar to those observed in other Spanish (Gómara et al. 2007; Vizcaino et al. 2011), European (Frederiksen et al. 2009), and Asian (Kim et al. 2012) populations but much lower than those reported in populations from North America (Foster et al. 2011; Herbstman et al. 2007a).

Some previous studies have considered the possible influence of total GWG in the accumulation of POPs in serum of pregnant women (Bradman et al. 2007; Castorina et al. 2011; Glynn et al. 2007), maternal milk (Daniels et al. 2010; Lignell et al. 2011), and newborns (Harley et al. 2011; Herbstman et al. 2007a; Stewart et al. 2000), but results have been inconsistent. Some of them found significant negative associations for PCBs, PBDEs, HCB, and 4,4´-DDT (Bradman et al. 2007; Glynn et al. 2007; Herbstman et al. 2007a; Lignell et al. 2011), whereas others found no association for PCBs, PBDEs, 4,4´-DDE, or β-HCH (Bradman et al. 2007; Castorina et al. 2011; Daniels et al. 2010; Glynn et al. 2007; Herbstman et al. 2007a). None of these previous studies considered the IOM GWG recommendations, and only two reported unadjusted relationships between PCBs, PBDEs (Herbstman et al. 2007a), and OCs (Bradman et al. 2007) and GWG categories different from the IOM recommendations. The potential modifier effect of prepregnancy BMI on GWG was not investigated in any study.

The multivariable results of the present study show an inverse association between GWG and POP levels in newborns when GWG is modeled as continuous variable (total kilograms), which is consistent with findings of earlier studies in pregnant women at late pregnancy (Bradman et al. 2007; Castorina et al. 2011; Glynn et al. 2007). A negative association between HCB and PCBs and GWG rate (% week) was found in a Swedish cohort of pregnant women who showed lower levels of PCBs and HCB before delivery (mean PCBs, 126 ng/g lipid and mean HCB, 23 ng/g lipid) after adjusting by age, year of sampling, and prepregnancy BMI (Glynn et al. 2007). In California, the CHAMACOS (Center for the Health Assessment of Mothers and Children of Salinas) cohort study observed a negative trend between GWG at 26 weeks gestation and PBDE levels in mothers (GM = 26 ng/lipid) adjusting for prepregnancy BMI among other potential confounders (Castorina et al. 2011; Harley et al. 2011). In the same California cohort, inverse associations between maternal OC levels and GWG were reported after adjusting for prepregnancy BMI (Bradman et al. 2007). In bivariate analyses, mothers from this cohort in the highest GWG category presented the lowest 4,4´-DDT levels.

In Baltimore, Maryland, PBDE but not PCB concentrations in cord serum were negatively associated with GWG adjusted by prepregnancy BMI and other determinants (Herbstman et al. 2007a). Newborns from mothers in the lowest GWG category showed the highest PBDE concentrations in univariate analyses. Finally, newborns whose mothers consumed PCB*-*contaminated fish from Lake Ontario did not show any association between PCB exposure and GWG in univariate analyses (Stewart et al. 2000).

In general, the observed associations between decreasing POP cord serum concentrations at increasing GWG may reflect a dilution of the circulating POPs in mothers, and consequently in their newborns, because of an expansion of maternal body fat stores and blood volume. Intake of POPs during the gestational period is therefore low compared with the amounts of these compounds accumulated in the maternal body over the lifetime. This is likely the case of women adhering to the recommended IOM GWG; they may deposit enough body fat during pregnancy to dilute POPs in their venous system or to prevent these compounds from incorporation into the blood.

Conversely, women not meeting the IOM weight recommendations might in fact lose weight during the formation of pregnancy-related organs such as the placenta, the amniotic fluid, the uterus, maternal breast tissue, or the fetus. Body weight loss has been associated with increasing plasma concentrations of POPs in adults (Chevrier et al. 2000). Inadequate level of maternal body fat may result in higher rates of mobilization of maternal fat stores in the last trimester of pregnancy (Haggarty 2010). This mobilization to meet the fetal demand may trigger the release of POPs into bloodstream, where they may cross the placenta barrier. In this respect, POP concentrations in adipose tissue of underweight mothers have been reported to be higher than in overweight or obese mothers (Kim et al. 2011).

Prepregnancy BMI showed only a negative association with HCB concentrations in cord serum of underweight mothers. Recent studies have reported both positive and negative associations of cord serum POP levels and maternal BMI (Dallaire et al. 2002; Herbstman et al. 2007a; Wolff et al. 2005b). In general, greater fat stores are considered to increase the body’s capacity to accumulate lipophilic contaminants (Wolff et al. 2005a), and the excretion rate of these contaminants is inversely proportional to BMI (Wolff et al. 2007). One of the most important modifiers of GWG is BMI at the start of pregnancy. Stratified results by prepregnancy BMI suggested that when underweight women increase GWG, they might release more POPs from their fat deposits into bloodstream and transfer them to their newborns, relative to other BMI categories. These findings might support the previous hypothesis that underweight women present higher rates of mobilization of maternal fat deposits during pregnancy (Haggarty 2010) and that their concentrations of POPs in adipose tissue are higher compared with overweight or obese mothers (Kim et al. 2011).

The multivariable model indicated that socioeconomic status, cumulative breastfeeding, and parity were not associated with cord serum levels after controlling for maternal age. Age was positively correlated with education level, parity, fish consumption, and cumulative breastfeeding. Older mothers had more children (30.4 years for nulliparous mothers vs. 33.2 years for multiparous) and longer history of lactation (30.6 years for absence of lactation vs. 33.9 years for breastfeeding mothers). Socioeconomic status was also associated with education level because mothers from higher classes tended to have university degrees (84%) and to be older. The lack of observed association between parity and cumulative breastfeeding may be related to the small proportion of multiparous women (36.9%) with a previous historial of lactation (29.1%) in the studied cohort.

Older mothers had newborns with higher OC concentrations, a trend that has been observed in other cohorts (Carrizo et al. 2006; Lackmann 2005). This age influence may also reflect a birth effect because mothers born in the early 1970s experienced higher environmental and dietary exposure to OCs during childhood than women born in the late 1980s (Glynn et al. 2007). In contrast, the relationship between PBDE levels and age is not clear; some studies have shown positive (Lignell et al. 2011), inverse (Herbstman et al. 2007a), or no association (Vizcaino et al. 2011). This lack of association may reflect the recent release of these compounds into the environment, which may involve a negative trend between cord serum concentration and maternal age.

In the present study, we found significant positive associations between cord serum concentrations of PCBs and PBDEs and maternal fish consumption, which is consistent with observations in foodstuffs (Gómara et al. 2006) and adult fish consumers (Domingo et al. 2008), including mothers (Llop et al. 2010). Higher-educated adults tend to have diets that are richer in fish (Darmon and Drewnowski 2008; Darnerud et al. 2006). In the present cohort, higher maternal education was observed to be related with lower 4,4´-DDE neonatal concentrations but not with lower PCBs, β-HCH, HCB, or PBDEs. These associations between maternal education and cord serum OC concentrations have not been observed in other studies (Vrijheid et al. 2012). No relationship between concentrations of 4,4´-DDE and food intake during pregnancy was identified in the present study. Thus, the origin of this association for 4,4´-DDE remains unclear.

## Conclusions

The present results suggest that GWG influences the accumulation of POPs in newborns. Neonatal concentrations of all POPs were lower in association with increasing GWG after adjustment for potential confounders. Other predictors such as maternal age, fish consumption during pregnancy, and educational level might be also relevant for the accumulation of POPs *in utero*. On average, mothers whose gestational weight gain was below IOM recommendations gave birth to newborns with higher POP concentrations than mothers who met or exceeded the weight gain recommendations (9%–30% higher than the recommended gain). Accordingly, the IOM recommendations for GWG during pregnancy may be beneficial for reducing POP exposures in newborns. These findings and previous results on the potential association between GWG and birth outcomes (Nohr et al. 2009) support the incorporation of GWG as covariate in epidemiological studies of effects of POPs on children’s health. IOM recommendations for GWG also have clinical value for primary care because women can thus reduce POP concentrations in their newborns.

## Supplemental Material

(757 KB) PDF
