# Supplementary material for: Gestational Weight Gain and Exposure of Newborns to Persistent Organic Pollutants
Source: Environ Health Perspect. 2014 May 2;122(8):873–9. doi: 10.1289/ehp.1306758 (PMC4123021; doi:10.1289/ehp.1306758)
Supplement: (757 KB) PDF [file ehp.1306758.s001.pdf]

*Environmental Health Perspectives* doi:10.1289/ehp.1306758

# Supplemental Material

# Gestational Weight Gain and Exposure of Newborns to Persistent Organic Pollutants

Esther Vizcaino, Joan O. Grimalt, Berit Glomstad, Ana Fernández-Somoano, and Adonina Tardón

**Table of Contents** **Page**

**Analytical methods** 2

**Table S1.** Distribution of gestational ages (weeks) at the time of the last weight measurements 3

**Table S2.** Observed descriptive statistics of weekly GWG in the INMA cohorts 4

**Table S3.** Cord serum concentrations of OCPs, PCBs, and PBDE congeners (ng/mL and ng/g lipid) in the study population 5

**Table S4.** Maternal concentrations of OCPs, PCBs and PBDE congeners (ng/mL [ng/g lipid]) in the study population 6

**Table S5.** GWG during pregnancy (using a measure >37.5 weeks of gestation, n=1476) *vs* estimated GWG using a previous measure 7

**Table S6.** Adjusted associations of GWG β (95% CI) with ln-transformed POPs concentrations (ng/g lipids) based on alternative models 8

**Table S7.** Adjusted associations β (95% CI) for ln-transformed individual PCB and BDE congeners (ng/g lipid) according to GWG category 9

**Figure S1.** Conceptual hypothesis of the study 10

**Figure S2.** Multivariate Tobit regression coefficients of individual ln-transformed PBDE congeners and GWG 11

**Figure S3.** Multivariate linear regression coefficients of ln-transformed individual PCBs congeners and GWG 12

**Reference** 13

## Analytical methods

Cord serum concentrations of α-HCH, β-HCH, γ-HCH, δ-HCH, HCB, PeCB, 2,4′-DDT, 4,4′-DDT, 2,4′-DDE, 4,4′-DDE, 2,4′-DDD, 4,4′-DDD, 7 PCB congeners (CB28, CB52, CB101, CB118, CB153, CB138 and CB180) and 14 PBDE congeners (BDE17, BDE28, BDE47, BDE66, BDE71, BDE85, BDE99, BDE100, BDE153, BDE154, BDE138, BDE183, and BDE190 and BDE209) were analyzed.

Briefly, 1 mL of serum was spiked with the surrogate standards tetrabromobenzene (TBB) and decachlorobiphenyl (CB209) and vortex stirred for 30 sec at 2,000 rpm. n-Hexane (3 mL) was added, followed by concentrated sulfuric acid (2 mL). After reaction, the mixture was stirred for 30 sec and the supernatant n-hexane phase was separated by centrifugation. The remaining sulfuric acid solution was re-extracted twice with 2 mL of n-hexane (each by 30 sec stirring and centrifugation). The combined n-hexane extracts (7 mL) were additionally cleaned with sulfuric acid (2 mL, stirring 30 sec). Then, the n-hexane phase was separated by centrifugation and reduced to a small volume under a gentle nitrogen stream. The extract was transferred to gas chromatography (GC) vials using four 25 μL rinses of isooctane. CB142, BDE118 (20 μL) and [$^{13}$C]-BDE209 (10 μL) were added as internal standards before injection. Organochlorine compounds (OCs) were determined by GC with electron capture detection. BDE congeners were analyzed by GC coupled to mass spectrometry in chemical ionization and negative ion recording.

Total cholesterol and triglycerides were determined by enzymatic methods in maternal and cord serum samples and total serum lipid concentrations were calculated as described elsewhere (Phillips et al. 1989).

**Table S1.** Distribution of gestational ages (weeks) at the time of the last weight measurements.

| Cohort, week k | Gestational age (weeks) | N[a] | %[b] |
|---|---|---|---|
| **Reference (n = 2413)** | | | |
| 18 | 16.5 - 19.5 | 4 | 0.2 |
| 21 | 19.5 - 22.5 | 22 | 0.9 |
| 24 | 22.5 - 25.5 | 7 | 0.3 |
| 27 | 25.5 - 28.5 | 17 | 0.7 |
| 30 | 28.5 - 31.5 | 47 | 1.9 |
| 33 | 31.5 - 34.5 | 225 | 9.3 |
| 36 | 34.5 - 37.5 | 615 | 25.5 |
| 39 | 37.5 - 42 | 1476 | 61.2 |
| **Asturias (n = 324)** | | | |
| 18 | 16.5 - 19.5 | 1 | 0.3 |
| 21 | 19.5 - 22.5 | 1 | 0.3 |
| 24 | 22.5 - 25.5 | 1 | 0.3 |
| 27 | 25.5 - 28.5 | 4 | 1.2 |
| 30 | 28.5 - 31.5 | 12 | 3.7 |
| 33 | 31.5 - 34.5 | 52 | 16.0 |
| 36 | 34.5 - 37.5 | 123 | 38.0 |
| 39 | 37.5 - 42 | 130 | 40.1 |

[a]Number of mothers in which the last weight measurement was determined at this gestational age. [b]Percent of mothers in which the last weight measurement was determined at this gestational age.

**Table S2.** Observed descriptive statistics of weekly GWG in the INMA cohorts.

| Week | N | Mean[a] | SD | Min | Max |
|---|---|---|---|---|---|
| **Reference (n=2413)** | | | | | |
| wGWG 18w | 915 | 0.230 | 0.172 | -0.38 | 0.88 |
| wGWG 21w | 1817 | 0.274 | 0.159 | -0.41 | 1.16 |
| wGWG 24w | 860 | 0.286 | 0.140 | -0.26 | 0.83 |
| wGWG 27w | 1093 | 0.311 | 0.137 | -0.19 | 0.78 |
| wGWG 30w | 1069 | 0.323 | 0.136 | -0.19 | 0.89 |
| wGWG 33w | 1696 | 0.328 | 0.130 | -0.16 | 0.96 |
| wGWG 36w | 1485 | 0.332 | 0.125 | -0.16 | 0.76 |
| wGWG 39w | 1476 | 0.346 | 0.126 | -0.19 | 0.98 |
| **Asturias (n=324)** | | | | | |
| wGWG 18w | 128 | 0.259 | 0.167 | -0.17 | 0.73 |
| wGWG 21w | 196 | 0.293 | 0.150 | -0.26 | 0.80 |
| wGWG 24w | 107 | 0.346 | 0.149 | -0.06 | 0.70 |
| wGWG 27w | 192 | 0.328 | 0.130 | -0.02 | 0.72 |
| wGWG 30w | 149 | 0.342 | 0.140 | -0.13 | 0.81 |
| wGWG 33w | 183 | 0.356 | 0.125 | 0.02 | 0.84 |
| wGWG 36w | 194 | 0.349 | 0.133 | -0.09 | 0.73 |
| wGWG 39w | 130 | 0.349 | 0.127 | 0.05 | 0.82 |

wGWG: weekly gestational weight gain, in kg/week. Only one measurement per interval was taken (the closest to the central value).

[a]Observed mean.

**Table S3.** Cord serum concentrations of OCPs, PCBs, and PBDE congeners (ng/mL and ng/g lipid) in the study population.

| Pollutants | 1/2LOD | >LOD(%) | GM (95% CI) ng/mL | Median ng/mL | P 90 ng/mL | Range ng/mL | GM (95% CI) ng/g lipid | Median ng/g lipid | P 90 ng/g lipid | Range ng/g lipid |
|---|---|---|---|---|---|---|---|---|---|---|
| 4,4´-DDT | 0.012 | 90 | 0.12 (0.10, 0.14) | 0.08 | 0.22 | nd-1.6 | 49 (42, 55) | 33 | 97 | nd-650 |
| 4,4´-DDE | 0.003 | 100 | 0.69 (0.61, 0.78) | 0.47 | 1.4 | nd-6.0 | 270 (240, 299) | 180 | 580 | nd-2000 |
| HCB | 0.005 | 98 | 0.17 (0.16, 0.19) | 0.13 | 0.37 | nd-1.0 | 67 (62, 73) | 50 | 140 | nd-430 |
| β-HCH | 0.0006 | 90 | 0.067 (0.059, 0.075) | 0.045 | 0.15 | nd-0.68 | 26 (23, 29) | 17 | 56 | nd-240 |
| PCB 138 | 0.012 | 92 | 0.11 (0.09, 0.14) | 0.08 | 0.18 | nd-4.0 | 45 (34, 55) | 31 | 68 | nd-1600 |
| PCB 153 | 0.007 | 98 | 0.14 (0.13, 0.15) | 0.13 | 0.25 | nd-0.87 | 56 (53, 60) | 47 | 96 | nd-320 |
| PCB 180 | 0.005 | 97 | 0.084 (0.079, 0.093) | 0.07 | 0.15 | nd-1.1 | 33 (30, 37) | 27 | 58 | nd-400 |
| ∑PCBs | --- | 99 | 0.45 (0.41, 0.48) | 0.38 | 0.72 | 0.068-4.4 | 175 (161, 190) | 150 | 280 | 24-1800 |
| BDE 47 | 0.0013 | 36 | 0.0049 (0.003, 0.007) | 0.0013 | 0.009 | nd-0.28 | 1.9 (1.2, 2.8) | 0.56 | 3.4 | nd-120 |
| BDE 99 | 0.0011 | 31 | 0.0056 (0.004, 0.007) | 0.0011 | 0.014 | nd-0.22 | 2.2 (1.6,2.9) | 0.5 | 5.4 | nd-90 |
| BDE 153 | 0.0006 | 43 | 0.003 (0.002, 0.05) | 0.0006 | 0.006 | nd-0.16 | 1.5 (0.95, 2.0) | 0.29 | 2.6 | nd-66 |
| BDE 209 | 0.0045 | 15 | 0.010 (0.008, 0.012) | 0.0045 | 0.020 | nd-0.17 | 4.1 (3.2, 5.0) | 1.9 | 7.4 | nd-61 |
| ∑BDEs | --- | 61 | 0.025 (0.019, 0.030) | 0.010 | 0.047 | nd-0.67 | 5.7 (5, 6) | 3.9 | 21 | nd-820 |

CI: confidence interval; GM: geometrical mean; LOD: Limit of detection; nd: not detected. P 90: $90^{th}$ percentile.

**Table S4.** Maternal concentrations of OCPs, PCBs and PBDE congeners (ng/mL [ng/g lipid]) in the study population.

| Pollutants | 1/2LOD | >LOD(%) | GM (95% CI) ng/mL | Median ng/mL | P 90 ng/mL | Range ng/mL | GM (95% CI) ng/g lipid | Median ng/g lipid | P 90 ng/g lipid | Range ng/g lipid |
|---|---|---|---|---|---|---|---|---|---|---|
| 4,4´-DDT | 0.012 | 80 | 0.12 (0.10, 0.14) | 0.10 | 0.24 | nd-2.3 | 26 (23, 30) | 19 | 45 | nd-219 |
| 4,4´-DDE | 0.003 | 100 | 2.0 (1.9, 2.3) | 1.4 | 4.6 | 0.23-20.6 | 405 (364, 447) | 243 | 782 | 35-3945 |
| HCB | 0.005 | 98 | 0.48 (0.44, 0.52) | 0.38 | 0.90 | 0.06-4.9 | 93 (86, 100) | 70 | 181 | 12.1433 |
| β-HCH | 0.0006 | 90 | 0.21 (0.19, 0.23) | 0.15 | 0.39 | nd-3.8 | 40 (36, 45) | 25 | 72 | nd-326 |
| PCB 138 | 0.012 | 100 | 0.23 (0.21, 0.25) | 0.20 | 0.38 | 0.05-1.6 | 45 (41, 48) | 39 | 75 | 9.05-322 |
| PCB 153 | 0.007 | 100 | 0.38 (0.35, 0.40) | 0.34 | 0.59 | 0.08-2.4 | 73 (69, 78) | 66 | 112 | 12.9-468 |
| PCB 180 | 0.005 | 99 | 0.28 (0.26, 0.31) | 0.25 | 0.44 | nd-3.0 | 55 (50, 60) | 48 | 86 | nd-596 |
| ∑PCBs | --- | 100 | 0.89 (0.83, 0.96) | 0.80 | 1.4 | 0.12-7.1 | 175 (162, 187) | 155 | 265 | nd-1387 |
| BDE 47 | 0.0013 | 22 | 0.005 (0.004, 0.007) | 0.0013 | 0.014 | nd-0.13 | 1.9 (1.2, 2.8) | 0.25 | 2.7 | nd-25 |
| BDE 99 | 0.0011 | 60 | 0.014 (0.012, 0.016) | 0.008 | 0.035 | nd-0.16 | 1.1 (0.82, 1.4) | 1.6 | 6.9 | nd-31 |
| BDE 153 | 0.0006 | 94 | 0.019 (0.016, 0.022) | 0.013 | 0.038 | nd-0.45 | 3.8 (3.2, 4.4) | 2.6 | 7.8 | nd-84 |
| BDE 209 | 0.0045 | 31 | 0.015 (0.012, 0.018) | 0.0045 | 0.043 | nd-0.16 | 3.1 (2.5, 3.6) | 0.9 | 8.7 | nd-39 |
| ∑BDEs | --- | 98 | 0.056 (0.051, 0.062) | 0.042 | 0.11 | nd-0.56 | 11 (10, 12) | 8.2 | 21 | nd-104 |

CI: confidence interval; GM: geometrical mean; LOD: limit of detection; nd: not detected; P90: $90^{th}$ percentile.

**Table S5.** GWG during pregnancy (using a measure >37.5 weeks of gestation, n=1476) *vs* estimated GWG using a previous measure.

| Variable | Time period | N | $R^2$ | Bias[a] | Me(abs RPD)[b] | RMSE | CV(RMSE)[c] |
|---|---|---|---|---|---|---|---|
| $twGWG^i_{36}$ | 34.5 - 37.5 weeks | 870 | 0.91 | 0.18 | 6.03 | 1.42 | 0.10 |
| $twGWG^i_{33}$ | 31.5 - 34.5 weeks | 1099 | 0.86 | 0.03 | 7.91 | 1.92 | 0.14 |
| $twGWG^i_{30}$ | 28.5 - 31.5 weeks | 635 | 0.80 | 0.02 | 9.54 | 2.30 | 0.17 |
| $twGWG^i_{27}$ | 25.5 - 28.5 weeks | 682 | 0.75 | 0.21 | 12.25 | 2.58 | 0.19 |
| $twGWG^i_{24}$ | 22.5 - 25.5 weeks | 564 | 0.67 | 0.48 | 13.41 | 2.93 | 0.21 |
| $twGWG^i_{21}$ | 19.5 - 22.5 weeks | 1148 | 0.59 | -0.06 | 16.00 | 3.40 | 0.25 |
| $twGWG^i_{18}$ | 16.5 - 19.5 weeks | 574 | 0.46 | -0.11 | 19.68 | 3.94 | 0.29 |

$R^2$: R-squared; Me (abs RPD): median absolute relative percent difference; RMSE: root-mean-square error; CV(RMSE): coefficient of variation of the RMSE.

[a]Bias = mean (estimated GWG – observed GWG). [b]Me(abs RPD) = 100 * median (|estimated $twGWG_{k39}$ – obs $twGWG_{39}$| / obs $twGWG_{39}$). [c]CV(RMSE) = $[(1/N)\ \Sigma_{k=1}^{N}\ (\text{estimated } twGWG_{k39} - \text{real } twGWG_{k39})^2]^{1/2}$ / mean (real $twGWG_{39}$).

**Table S6.** Adjusted[a] associations of GWG β (95% CI) with ln-transformed POPs concentrations (ng/g lipids) based on alternative models**.**

| POP | Main model | Women with no weight measurements <28.5 weeks excluded (n=7) | Maternal POPs concentrations excluded | Relative difference in newborn concentrations compared with maternal concentrations (X) |
|---|---|---|---|---|
| ΣPCBs | -0.01 (-0.02, -0.002) | -0.01 (-0.02, -0.004) | -0.02 (-0.03, -0.008) | -0.008 (-0.02, -0.01) |
| ΣBDEs | -0.02 (-0.05, 0.008) | -0.02 (-0.04, 0.01) | -0.01 (-0.03, 0.007) | -0.003 (-0.02, 0.01) |
| 4,4´-DDE | -0.016 (-0.03, -0.003) | -0.017 (-0.03, 0.004) | -0.02 (-0.04, -0.002) | -0.008 (-0.01, -0.002) |
| 4,4´-DDT | -0.013 (-0.03, 0.005) | -0.016 (-0.03, 0.001) | -0.018 (-0.04, 0.0008) | -0.007 (-0.05, 0.04) |
| HCB | -0.012 (-0.03, 0.001) | -0.014 (-0.03, -0.001) | -0.012 (-0.03, 0.004) | -0.009 (-0.02, -0.002) |
| β-HCH | -0.03 (-0.06, -0.003) | -0.03 (-0.06, -0.004) | -0.06 (-0.1, -0.03) | -0.01 (-0.02, -0.004) |

[a]Unless otherwise specified, models for each POP included the following covariates. ΣPCBs: pre-pregnancy BMI, age, total fish consumption, maternal ΣPCB concentrations. ΣBDEs: pre-pregnancy BMI, total fish consumption, maternal ΣBDE concentrations. 4,4´-DDE: pre-pregnancy BMI, age, maternal education, maternal 4,4´-DDE concentrations. 4,4´-DDT: pre-pregnancy BMI, age, maternal 4,4´-DDT concentrations HCB: pre-pregnancy BMI, age, total fish consumption, maternal HCB concentrations. β-HCH: pre-pregnancy BMI, age, total fish consumption, maternal. β-HCH concentrations.

**Table S7.** Adjusted[a] associations β (95% CI) for ln-transformed individual PCB and BDE congeners (ng/g lipid) according to GWG category.

| POP | Inadequate GWG (n = 81) [b] | Excessive GWG (n = 135) [b] |
|---|---|---|
| BDE47 | 0.46 (-0.17, 1.1) | -0.23 (-0.82, 0.35) |
| BDE99 | 0.45 (-0.48, 1.4) | -0.28 (-1.2, 0.58) |
| BDE153 | 0.04 (-0.64, 0.57) | -0.36 (-0.91, 0.18) |
| BDE209 | 1.26 (0.006, 2.5) | 0.51 (-0.63, 1.66) |
| PCB138 | 0.16 (-0.013, 0.33) | 0.07 (-0.08, 0.22) |
| PCB153 | 0.15 (0.02, 0.30) | -0.02 (-0.15, 0.11) |
| PCB180 | 0.13 (-0.02, 0.29) | -0.16 (-0.29, -0.01) |

[a]Models of PCB congeners adjusted by: pre-pregnancy BMI, age, total fish consumption, maternal PCB congener concentrations. Models of PBDE congeners adjusted by: pre-pregnancy BMI, total fish consumption, maternal PBDE congener concentrations. [b]Reference group is Recommended GWG (n=108).

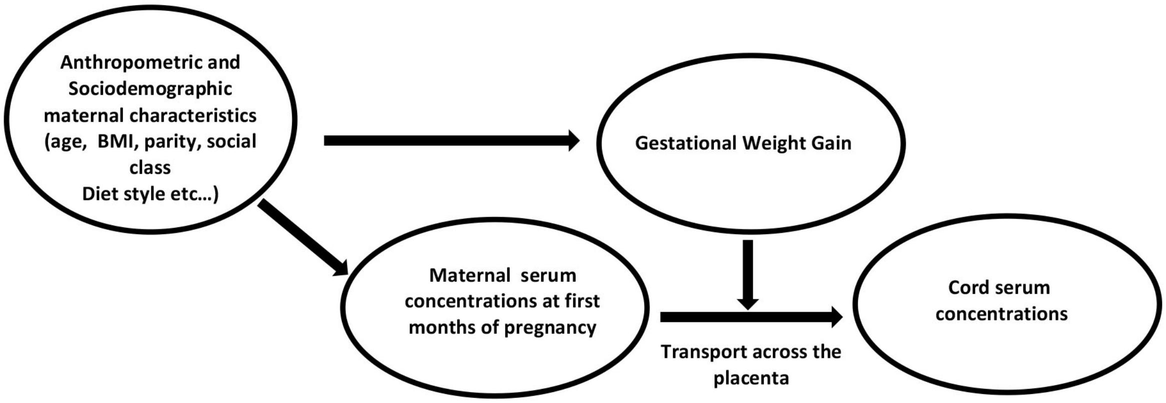


**Figure S1.** Conceptual hypothesis of the study.

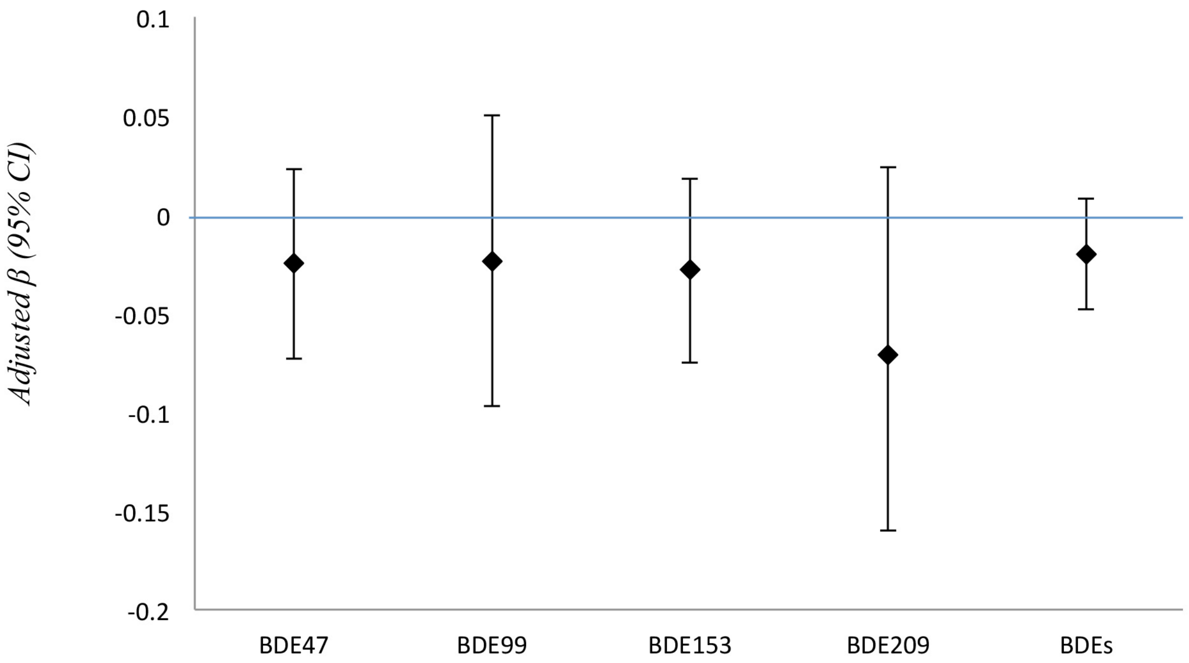


**Figure S2.** Multivariate Tobit regression coefficients of individual ln-transformed PBDE congeners and GWG.

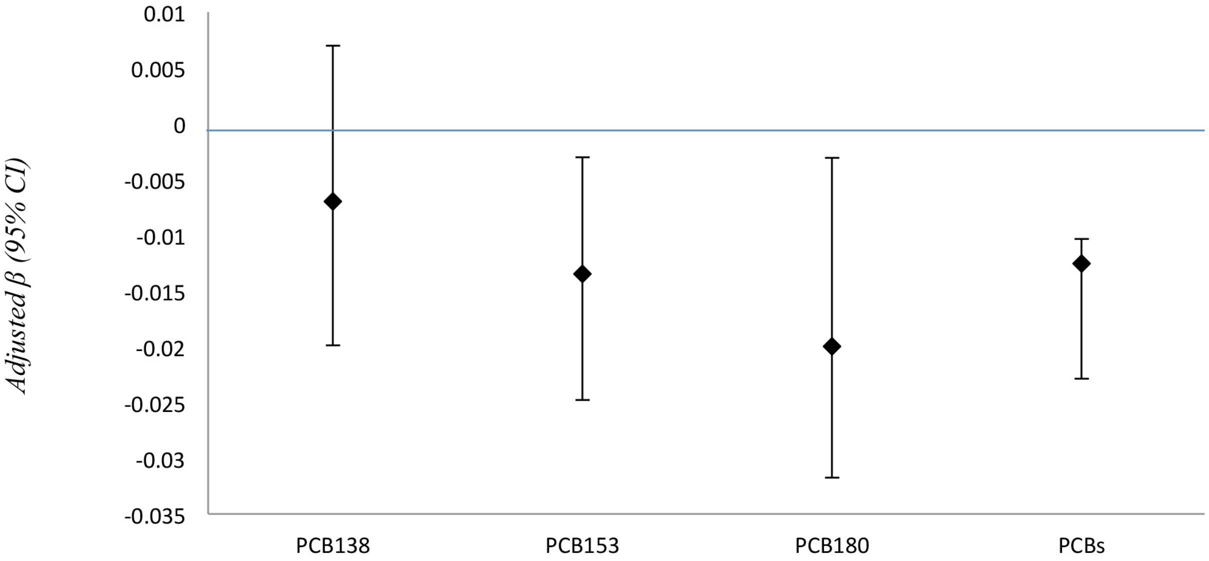


**Figure S3.** Multivariate linear regression coefficients of ln-transformed individual PCBs congeners and GWG.

## Reference

Phillips DL, Pirkle JL, Burse VW, Bernert Jr JT, Henderson LO, Needham LL. 1989. Chlorinated hydrocarbon levels in human serum: Effects of fasting and feeding. Arch Environ Contam Toxicol 18(4):495-500.
